# Supplementary material for: Preventive Effects of Anthocyanins from Lycium ruthenicum Murray in High-Fat Diet-Induced Obese Mice Are Related to the Regulation of Intestinal Microbiota and Inhibition of Pancreatic Lipase Activity
Source: Molecules. 2022 Mar 26;27(7):2141. doi: 10.3390/molecules27072141 (PMC9000451; doi:10.3390/molecules27072141)
Supplement: Supplementary file 1 [file molecules-27-02141-s001.zip › molecules-1627055-supplementary.pdf]

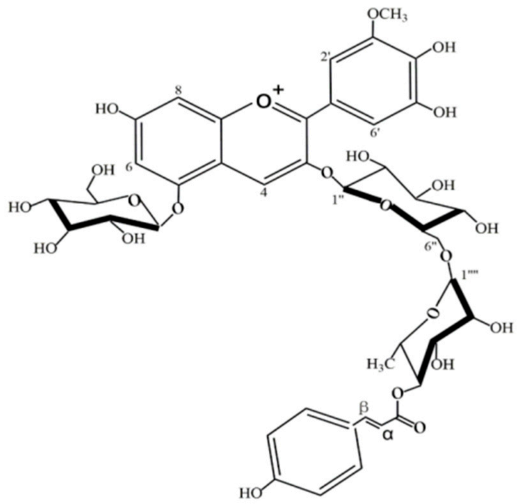

(**a**)

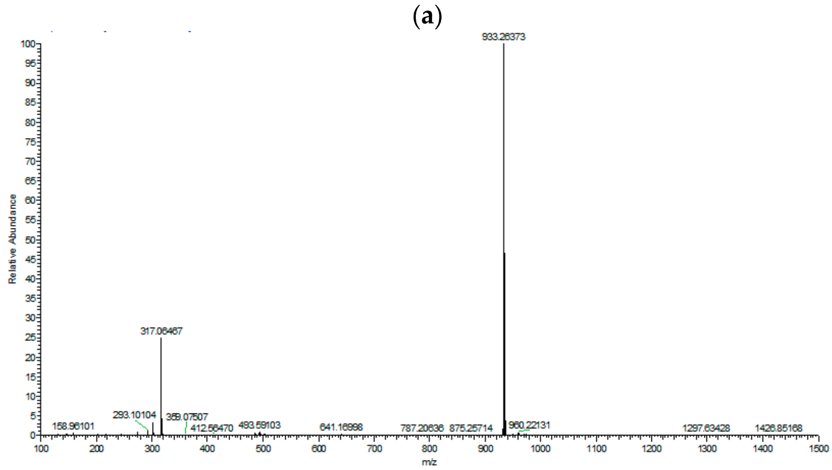

(**b**)

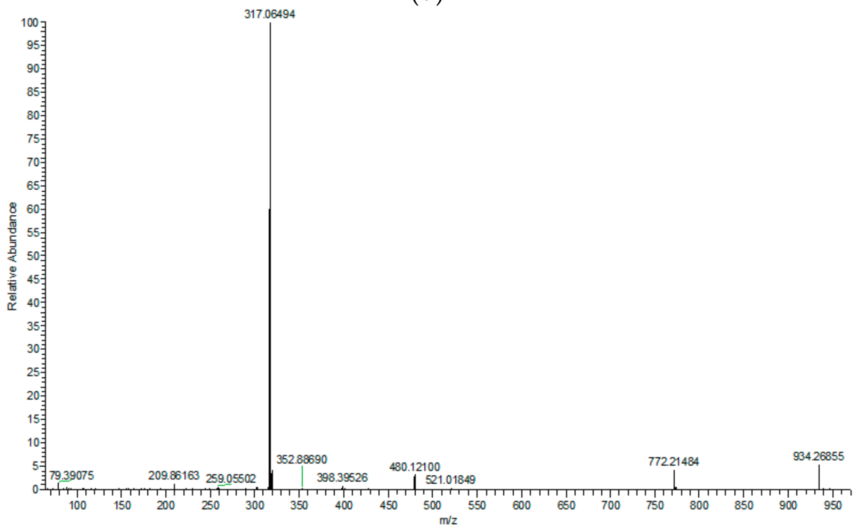

(**c**)

Figure S1. The main compound anthocyanin from *Lyciumruthenicum* Murray. (**a**) The chemical structure.(**b**)MS ofthe main compound anthocyanin. (**c**) MS^2 of the main compound anthocyanin.
